# Applicability of a semiautomated volumetric approach (5D CNS+™) for detailed antenatal reconstruction of abnormal fetal CNS anatomy

**DOI:** 10.1186/s12880-022-00888-1

**Published:** 2022-09-02

**Authors:** Amrei Welp, Michael Gembicki, Christoph Dracopoulos, Jann Lennard Scharf, Achim Rody, Jan Weichert

**Affiliations:** Division of Prenatal Medicine, Department of Obstetrics and Gynecology, University Hospital of Schleswig-Holstein, Campus Luebeck, Ratzeburger Allee 160, 23538 Luebeck, Germany

**Keywords:** Brain, 3D ultrasound, Semiautomatic reconstruction, Anomalies, Central nervous system

## Abstract

**Background:**

The aim of this study was to evaluate the accuracy and reliability of a semiautomated volumetric approach (5D CNS+™) when examining fetuses with an apparent abnormal anatomy of the central nervous system (CNS).

**Methods:**

Stored 3D volumes extracted from a cohort of > 1.400 consecutive 2nd and 3rd trimester pregnancies (range 15–36 gestational weeks) were analyzed using the semiautomatic software tool 5D CNS+™, enabling detailed reconstruction of nine diagnostic planes of the fetal brain. All 3D data sets were examined and judged for plane accuracy, the need for manual adjustment, and fetal CNS anomalies affecting successful plane reconstruction.

**Results:**

Based on our data of 91 fetuses with structural cerebral anomalies, we were able to reveal details of a wide range of CNS anomalies with application of the 5D CNS+™ technique. The corresponding anatomical features and consecutive changes of neighboring structures could be clearly demonstrated. Thus, a profound assessment of the entire altered CNS anatomy could be achieved in nearly all cases. The comparison with matched controls showed a significant difference in volume acquisition (*p* < 0.001) and in need for manual adjustment (*p* < 0.001) but not in the drop-out rates (*p* = 0.677) of both groups.

**Conclusion:**

5D CNS+™ is applicable in the majority of cases with brain lesions and constitutes a reliable tool even if the integrity of the fetal CNS is compromised by structural anomalies. Using volume data that were acquired in identical cutting sections needed for conventional biometry allows for detailed anatomic surveys grossly independent of the examiner’s experience.

**Supplementary Information:**

The online version contains supplementary material available at 10.1186/s12880-022-00888-1.

## Background

Although the central nervous system (CNS) was the first fetal anatomic structure whose pathology was successfully visualized in utero more than 100 years ago and although anencephaly was the first fetal anomaly to be diagnosed prior to viability using ultrasound in the 1970s, CNS abnormalities comprise one of the most common causes of referral for prenatal assessment that evade early diagnosis [1–3]. Their overall prevalence has recently been estimated to be 9.8 per 10,000 live births or potentially higher with newer imaging technologies, as discussed below [4, 5]. The apparent continuing trend of improved detection of a subset of CNS anomalies may also be attributed to increased prenatal screening. On the other hand, the increasing prevalence of different entities may reflect increases in potential risk factors, although more detailed information and the impact of confounding cofactors are mostly lacking.

The majority of severe congenital anomalies of the fetal CNS can be identified prenatally by applying a systemic, protocol-based ultrasound survey carefully performed by a knowledgeable and experienced examiner following established guidelines as previously shown (overall accuracy of 98% in cases with multiple anomalies and 81% in those with isolated lesions) [6]. Nevertheless, the detection rates of fetal brain lesions in an unselected population remain unsatisfactory, which may in part be explained by the fact that differentiation and growth of the fetal CNS is not completed even until after birth. Moreover, many complex CNS lesions develop throughout gestation and often become apparent only later in pregnancy. The implications of delayed detection and/or misdiagnoses of abnormal brain anatomy can be profound and per se constitute a source of anxiety, as these anomalies often carry a poor prognosis and might also be related to malformation syndromes that further deteriorate the individual outcome both in utero and after birth [7, 8].

In this regard, the diagnostic value of a three-dimensional (3D) all-embracing examination of the entire fetal brain has been clearly demonstrated in the literature [9, 10]. However, applying 3D ultrasound (3DUS) to obtain exact diagnostic planes for a detailed neurosonogram (as claimed by national and international guidelines) is highly operator-dependent, correlates to the examiner’s expertise, and can be considerably hampered by fetal or maternal factors (e.g., unfavorable fetal position in utero, high maternal body mass index, or fetal movements). Recent studies have shown that a semiautomated volumetric approach has the potential to overcome these limitations, since this workflow-based technique (5D CNS+™) has been proven to be an accurate and reliable technique for fetal neurosonography [8, 11–14].

The primary objective of this study was to evaluate both the validity and accuracy of 5D CNS+™ in proper reconstruction of all diagnostic planes in the case of an abnormal fetal CNS anatomy. The findings could constitute the basis of ideal interdisciplinary counseling and may in turn provide answers to the most likely pre- and postnatal course of the disease.

## Methods

### Study population

Three-dimensional volume datasets from all fetuses with an apparently abnormal CNS anatomy obtained by two operators with high expertise in performing prenatal ultrasound and acquired between April 2015 and December 2020 at a single referral center were included in this study. All examinations were performed transabdominally during a targeted anatomical survey using a Samsung WS80A Elite and HERA W10 ultrasound system (Samsung Medison, Seoul, Korea) equipped with a 1–8 MHz curved transducer (S-Vue™ Transducer CV1-8A). The referral base comprised a mixed-risk cohort (women at either high or low risk for fetal abnormalities). The study protocol was approved by the institutional ethics committee, and informed consent was obtained. To ensure a comprehensive evaluation of the CNS anatomy, only 3D volumes recorded between the 15th and 36th gestational weeks that met sufficient quality requirements were included. Eligibility criteria further included all cases affected by major structural CNS anomalies (Table 3). Exclusion criteria were: (1) minor lesions that not necessarily require further diagnostic or therapeutic treatment; (2) inadequate image quality; (3) incomplete capture of the skull/CNS during volume acquisition; (4) inability to apply the 5D CNS™ tool. A matched control group (3:1 matching for both maternal and pregnancy variables—e. g., maternal BMI, parity, gestational age, amniotic fluid index) was created to assess whether, in the case of CNS anomalies, more volumes had to be acquired per patient or whether manual readjustments of the cutting planes (incl. dropout rate) were necessary more frequently.

### Software application and volume processing

**Fig. 1:**
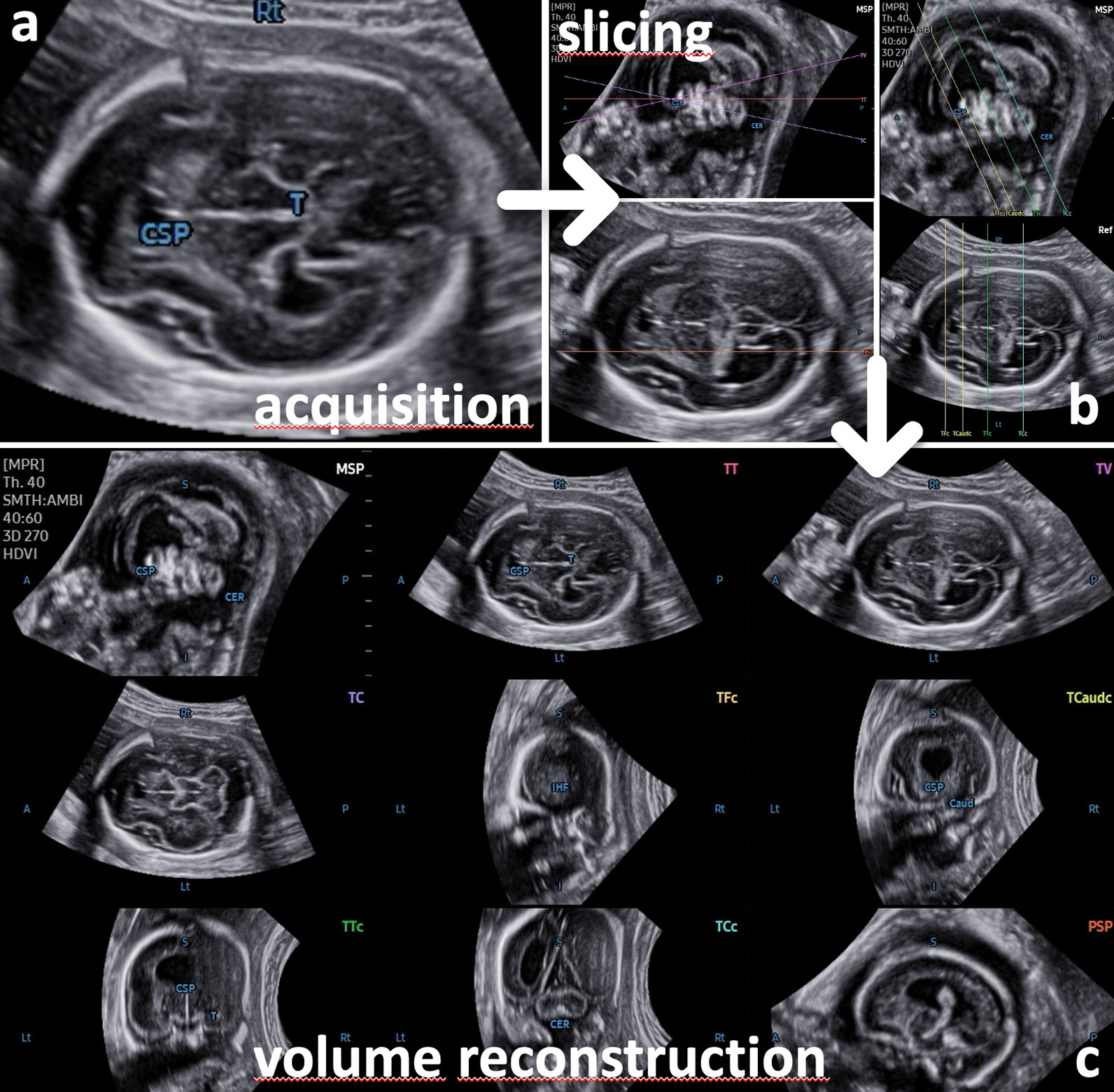
The transthalamic diagnostic plane (recommended for biparietal diameter quantification) is needed for proper volume acquisition. The part of the frontal lobe is not sufficiently delineated, and an enlargement of the lateral ventricle is suspected. For further assessment, additional views are necessary. Application of the 5D CNS+™ algorithm automatically reslices the volume data set in predefined diagnostic planes as needed for a complete neurosonogram. The reconstructed planes show abnormal CNS morphology found in errors of ventral induction (e.g., semilobar holoprosencephaly)

Originating from an axial view of the fetal skull, which corresponds to the transventricular plane, all 3D volumes were acquired in the absence of fetal movements and maternal breathing (for further details see Table 1; Fig. 1; Additional file 1: Video clip 1). In the resulting triplanar view, the orthogonal planes were reoriented to align the falx cerebri horizontally (A and B plane). Then, to run the 5D CNS+™ tool, two reference points were placed manually (the 1st seed placed in the middle of the thalami and the 2nd seed placed in the cavum septi pellucidi) followed by the automatic reconstruction of the nine diagnostic planes of the CNS as described previously. In the case of an apparently absent cavum septi pellucidi (CSP), the second point was placed on the midline or close to the estimated level of the CSP when gross forebrain anomalies were present (Additional file 2: Video clip 2).

**Table 1:** Recommended steps for appropriate volume acquisition and postprocessing using 5D CNS+™ (adapted from Dall’Asta et al. 2019 and Abuhamad 2005) [65, 66]

	General considerations	Specific recommendations
Patient selection	All pregnant women are theoretically eligible	Limitations such as high maternal body mass index, fetal movement, and unfavorable fetal position may occur
	No informed consent needed	
	Volumetric approach may be regularly included in anatomic survey	
	Ideal gestational age starting from 20 to 34 completed weeks (occasionally even at earlier GA)	Consider that anatomic structures may have not yet fully developed before 20 completed weeks
General machine settings	Presets need to be adjusted at a higher contrast and smaller dynamic range	An initial orienting 2D evaluation of the intracranial anatomy using the same image settings is mandatory (ideally in advance of the volume acquisition)
Volume acquisition	Insonation for 3D transabdominal volume acquisition	A transthalamic axial plane is a prerequisite for proper volumetry
		Other scanning planes potentially suitable for 3D brain assessment are not applicable
	Region of Interest (ROI) position and size	ROI should capture the entire contour of the fetal head (the box boundaries should be placed outside the skull)
	Scanning angle (sweep width of the 3D acquisition) and quality	Scanning angle needs to be adjusted according to the GA (between 60 and 85°), scan quality needs to be highest (‘extreme)
		Visualization of the cerebellum is crucial
Intermediate steps	Manipulation of the triplanar volume display along the x, y, and z-axis	The falx cerebri needs to be orientated horizontally in both the a and b planes
	Application of 5D CNS+™ and following the onscreen pictograms	Two reference marks need to be placed:
		1st seed between the rostralmost third of the thalami,
		2nd seed central in the cavum septi pellucidi
Reconstruction	Automatic reslicing of the volume to generate nine diagnostic planes for a complete neurosonogram	Generation of the entire template takes approximately 3–5 s
		Evaluation of all planes in a single template or grouped for axial, coronal, and sagittal planes separately
Postprocessing	Optimization of diagnostic plane alignment	If needed manual plane adjustment (plane by plane)
	Adjustment of biometric measurement	Manual correction of the calipers for exact biometric assessment of the CNS
		Integration of these measures into the structured biometric report

All statistical tests were run through Sigmaplot (Version 12.0, SyStat, USA). To compare the abnormal cases with match controls, the paired t-test (Normality Test Shapiro–Wilk) and Wilcoxon Signed Rank Test were used. *p* < 0.05 was considered to be statistically significant.

## Results

A total of 79 cases were enrolled in the final analysis after excluding 11 cases with minor cerebral anomalies (e.g., isolated plexus cysts, borderline ventricle diameter, or dolichocephaly) because these anatomic alterations did not meet the inclusion criteria. In one case, the semiautomatic reconstruction of all planes failed in a fetus affected by a giant encephalocele and was therefore excluded from final analysis.

The mean gestational age at the time of examination was 23.7 weeks (ranging from 16.6 to 35.1 weeks), the mean maternal age was 31.2 years (ranging from 19 to 44 years), and the mean maternal body mass index was 26.9 (ranging from 17.6 to 47.5 kg/m^2^) (see Table 2).

**Table 2:** Birth and maternal characteristics

Birth and maternal characteristics	Value/mean [range]
Maternal age at diagnosis (years)	31.8 [20–44]
Termination of pregnancy (TOP/n)	40
stillbirth (n)	3
Live-born infants (n)	35
Spontaneous vaginal (n)	16
Primary cesarean section (n)	11
Secondary cesarean section (n)	8
> 37 weeks of pregnancy (n)	24
< 37 weeks of pregnancy (n)	9
Gestational age at diagnosis (weeks)	22.2 [13.6–35.0]
Gestational age at TOP (weeks)	22.4 [17.6–32.3]
Gestational age at delivery (weeks)	35.3 [29.3–42.1]

**Fig. 2:**
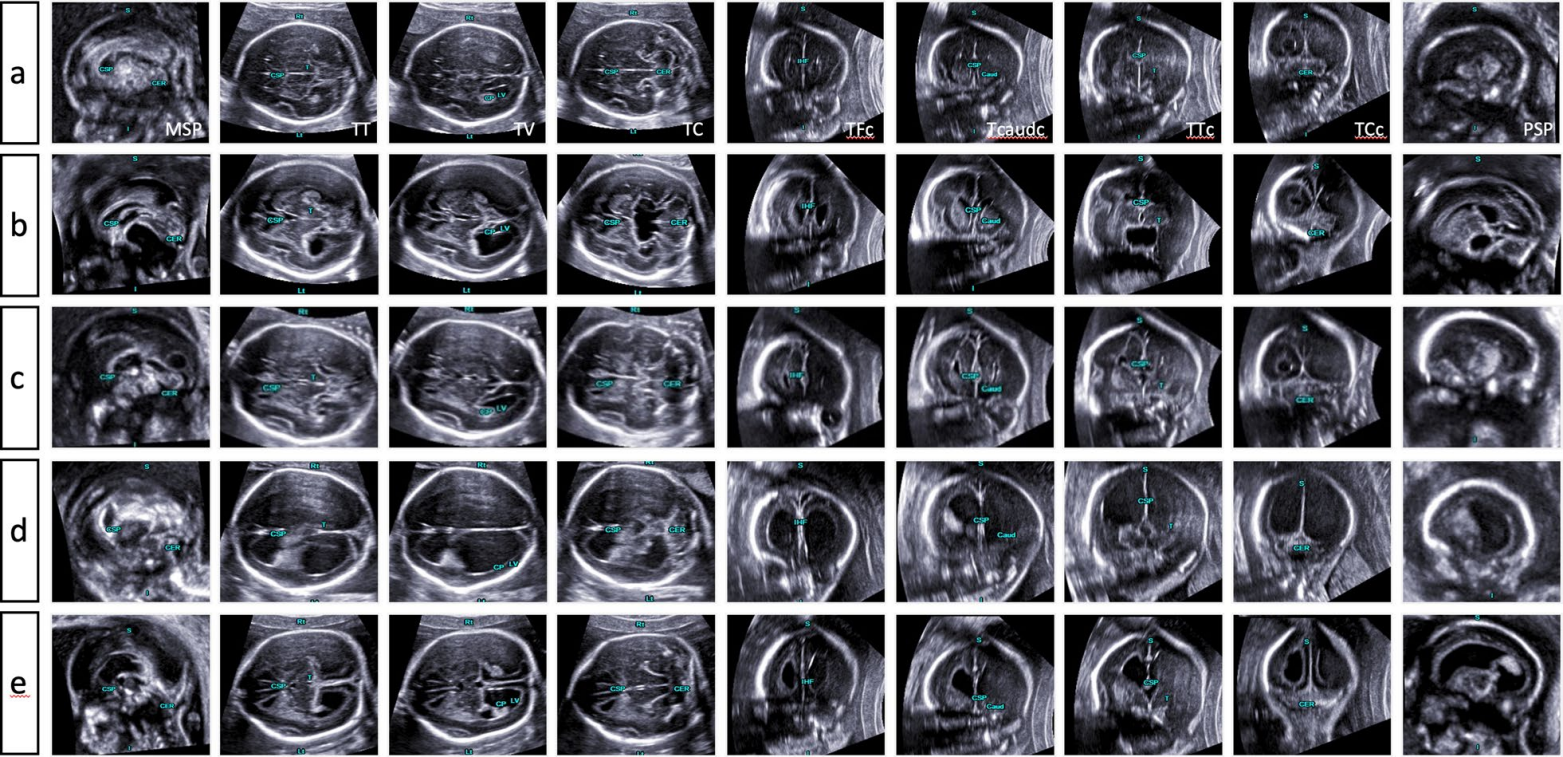
Comparative alignment of complete neurosonograms comprising nine diagnostic planes of normal (**a**) and abnormal CNS anatomy (**b**–**e**). Panel b demonstrates a cystic lesion located in the midline. The information gained from different cutting sections shows a slight enlargement of the 3rd ventricle (and reduced interthalamic adhesion diameter) but normal appearance of the aqueduct seen in the midsagittal and transventricular and transcerebellar planes. The lesion is more caudally located, expanding symmetrically toward the median border of the lateral ventricles, both of which are slightly enlarged, suggesting a functional obstruction of cerebrospinal fluid (CSF) drainage via the left and right foramen of Monro. Panels c-e depict varying degrees of ventricular enlargement caused by different underlying causes. Agenesis of corpus callosum with colpocephaly (panel c), note the absent cavum septi pellucidi seen in transthalamic and anterior coronal planes; the patent aqueduct in the midsagittal and axial planes as well as the steer horn/bull’s head appearance of the anterior horns displayed in the transcaudate cutting section. Panel d shows features of occlusive hydrocephaly clearly emphasized in nearly all diagnostic planes and most likely caused by aqueductal stenosis (dilated 3rd ventricle and nonvisualization of the sonolucent aqueduct in midsagittal and axial planes). Panel e illustrates abnormal intracerebral findings attributed to a Chiari II malformation as a sequela from spina bifida aperta (descent of the tonsils and abnormal bowing of cerebellum in midsagittal and transcerebellar planes). There was also a marked dilatation of the lateral ventricles seen in all planes

**Fig. 3:**
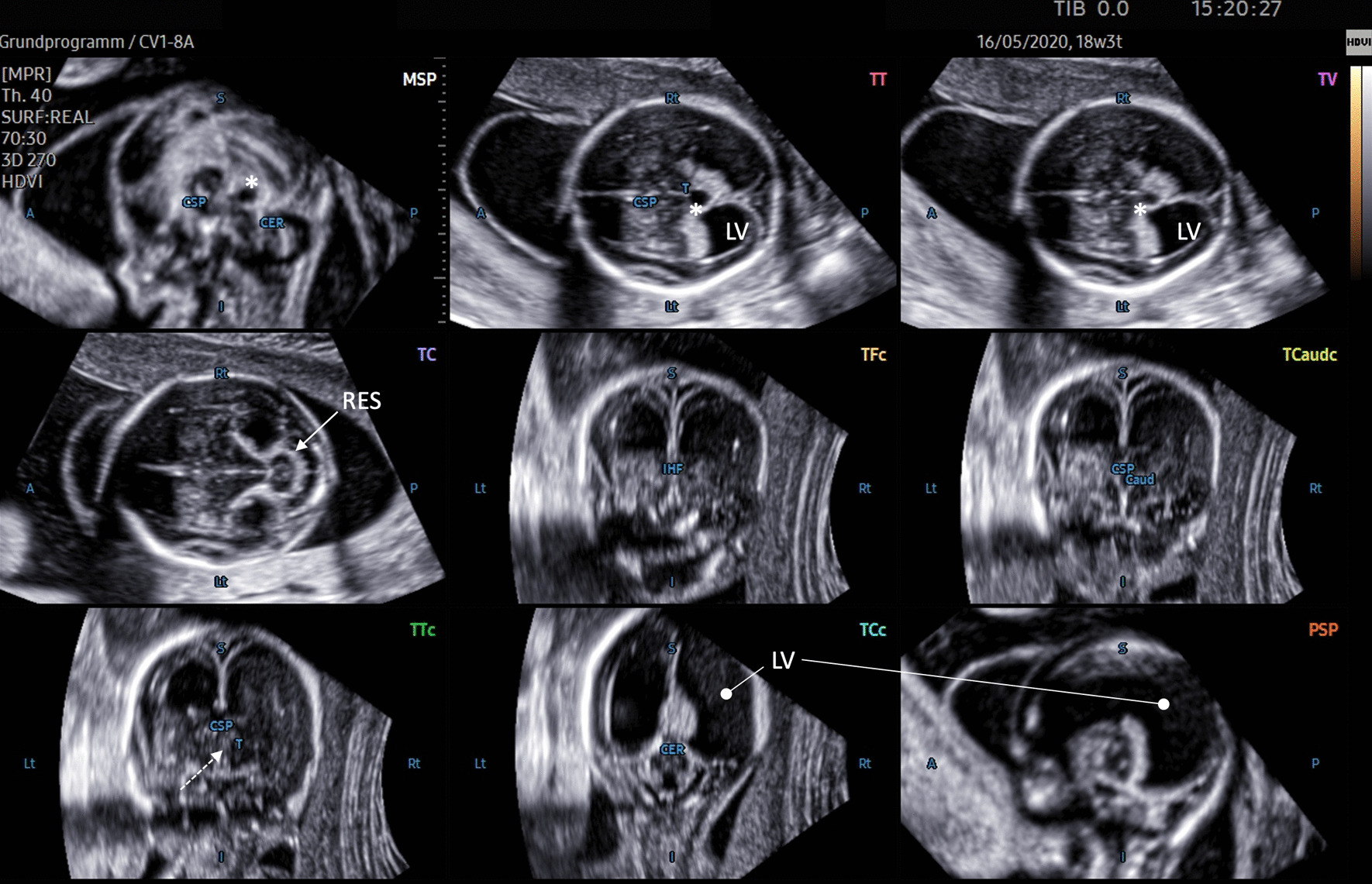
Nine-image template after 5D CNS+™ application depicting abnormal CNS anatomy of a dichorial twin gestation at 18 completed weeks. The transthalamic plane (TT; acquisition plane) shows enlarged lateral ventricles (LV) and a fluid-filled area (*) in the midline (also seen in the sagittal and transventricular (TV) cutting sections), most likely representing a dilated suprapineal recessus. Turricephaly was clearly displayed in the sagittal planes. The aqueduct of Sylvius cannot be distinguished in either the sagittal or transcerebellar plane (TC), which accomplishes the clinical picture of an obstructed liquor circulation. The transverse diameter of the cerebellum is small, suggesting severe hypoplasia and fusion of the hemispheres, as found in rhombencephalosynapsis (RES; solid arrow). The coronal transthalamic plane (TTc) reveals thalamic fusion (dotted arrow)

**Fig. 4:**
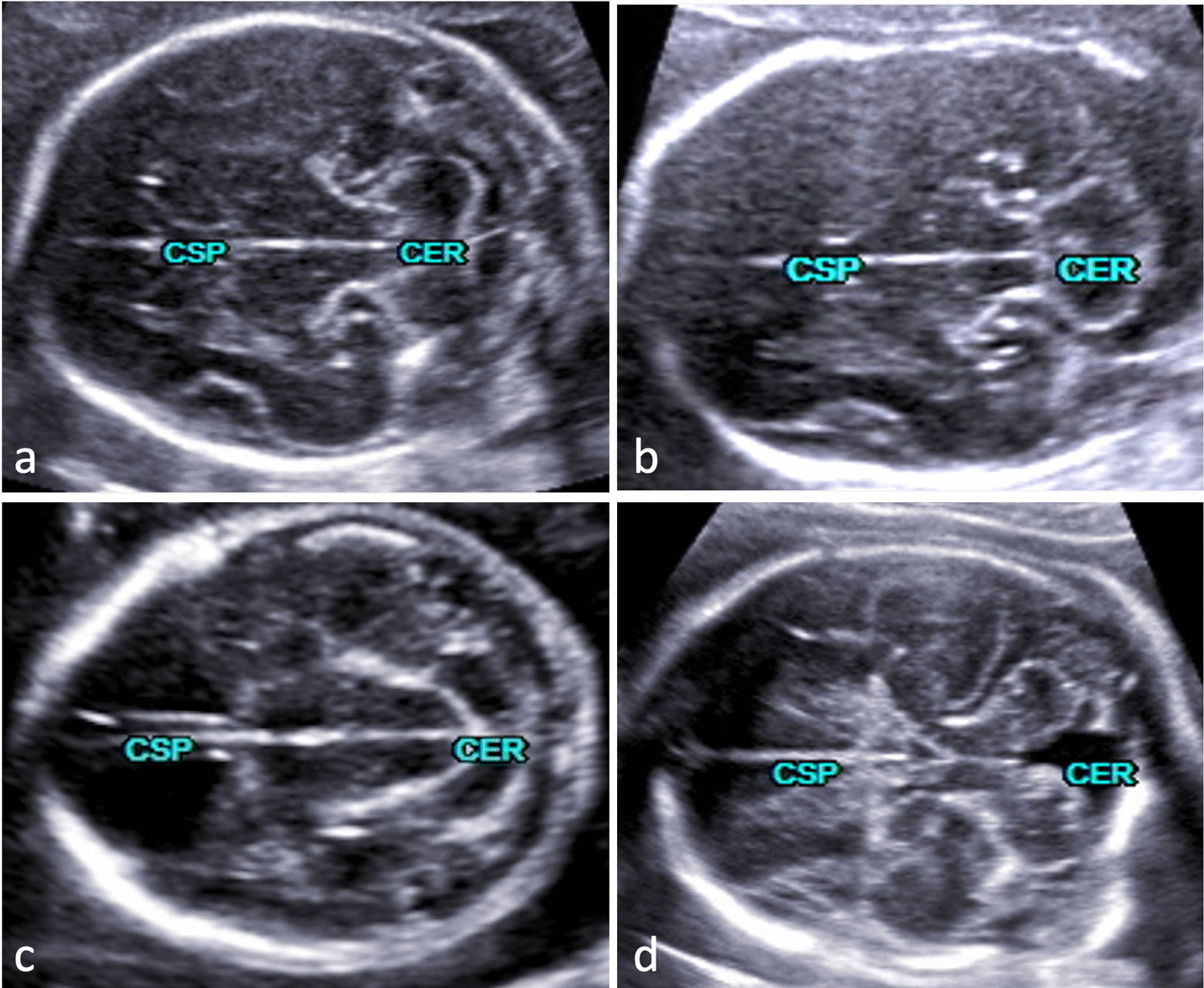
Transcerebellar plane depicting different cerebellar appearances from normal (**a**) to abnormal (**b**–**d**). The latter findings are part of gross intracranial pathology that needs further planes for delineating additional anomalies and establishing the final diagnosis. The rhombencephalosynapsis seen in panel b should necessarily stress an assessment of the ventricular system including the aqueduct of Sylvius (see also Fig. 3). In this particular case, aqueductal stenosis and triventricular enlargement were confirmed. An obstructed CSF pathway resulting in dilated lateral ventricles seen in all diagnostic planes underscored the impression of a Chiari II malformation (panel c). The fetus in panel d had vermian hypoplasia referred to as Dandy-Walker malformation

**Fig. 5:**
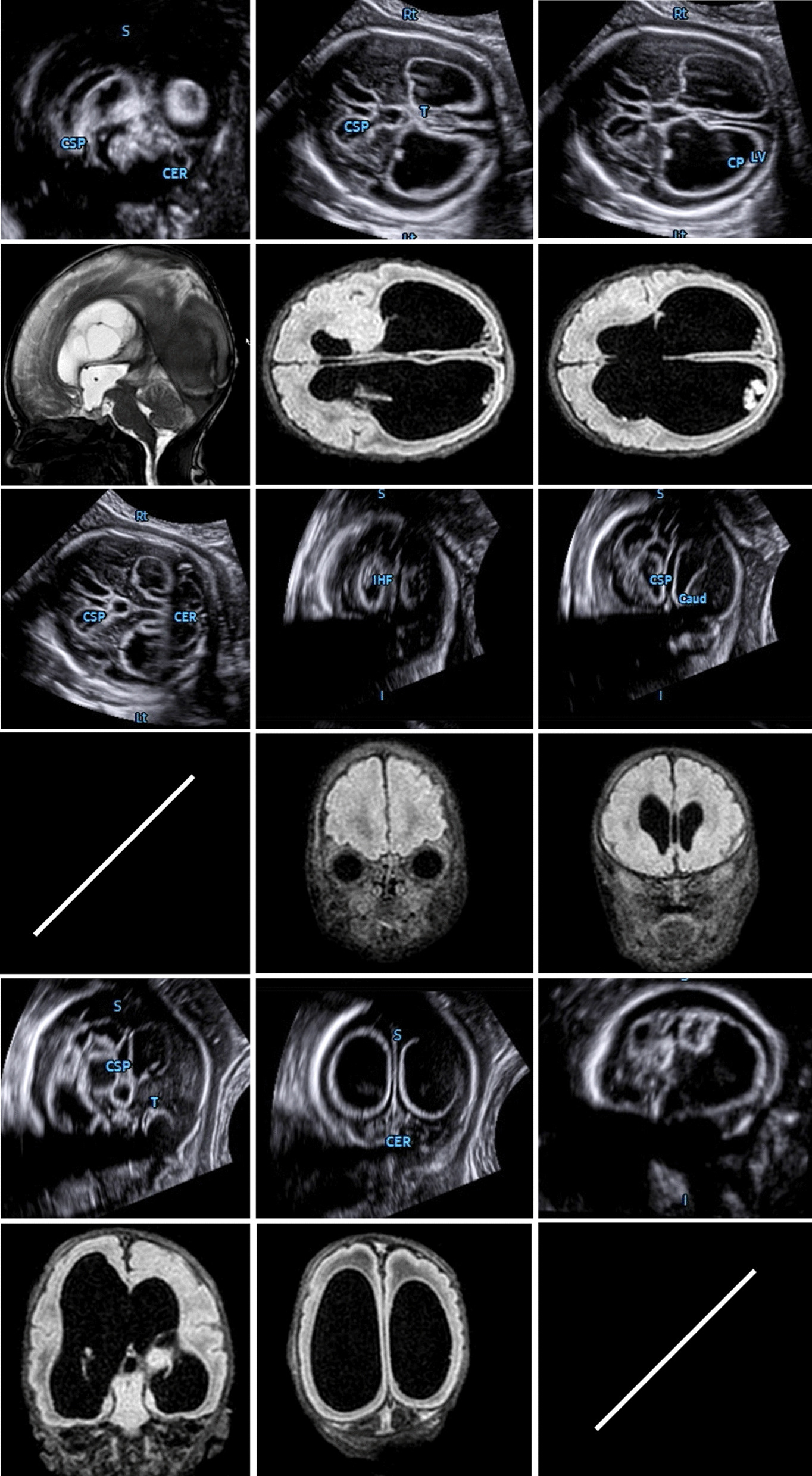
Intraventricular hemorrhage delineated using 5D CNS+™ in utero at 35 gestational weeks and correlated to the corresponding MR appearance on the 1st day after cesarean section. Note the severe hydrocephalus and the echogenic outlining of the ventricles. The blood clots predominantly seen in the axial and anterior coronal planes during prenatal imaging were markedly degraded in size or could not be reproduced postnatally. However, severe asymmetric enlargement of the ventricular system remained, and the cerebral cortex was thinned, corroborating the prenatal findings. MR images that correspond exactly for both the transcerebellar and parasagittal planes from 5D CNS+™ reconstruction could not be assigned due to the predetermined oblique cutting section.

A variety of major CNS anomalies were present in the population enrolled in this study (see Figs. 2, 3, 4, 5). Regarding the predominant defect, the included fetuses comprised those with neural tube defects (n = 22), ventriculomegaly (n = 15), hydrocephaly (n = 13), holoprosencephaly (n = 6), cystic lesions (n = 6), Dandy-Walker malformation (n = 4), corpus callosum agenesis (n = 6), rhombencephalosynapsis (n = 2), and mega cisterna magna (n = 2). One fetus had a vascular malformation of the circle of Willis, one had an intracranial hemorrhage, and another suffered from tuberous sclerosis, as described in Table 3. In ten fetuses, an additional chromosomal aberration was identified. A subanalysis of our data reviewed the occurrence of CNS anomalies in euploid and aneuploid fetuses. In our cohort, occlusive lesions as well as midline and fossa posterior anomalies could be observed in both groups, whereas spinal and solely cystic lesions exclusively occurred in euploid fetuses (see Table 1). Fifty-nine percent (40 of 67 cases) were terminated on parental request, and the mean gestational age at termination of pregnancy (TOP) was 22.2 weeks (range 17.6–32.3). In 33 cases, the parents opted for continuation of the pregnancy. The mean gestational age at delivery was 35.3 weeks (range 29.3–42.1). Three fetuses were stillborn.

**Table 3:** Clinical classification of CNS anomalies

CNS anomaly	n
Isolated ventriculomegaly	15
Occlusive lesions	13
Neural tube defects	22
Midline defects	12
Posterior fossa anomalies	8
Vascular malformations	1
Tumors/cysts	6
Intracranial hemorrhage	1
Tuberous sclerosis	1

To ascertain fetal CNS anomalies that might hamper proper application of the 5D CNS+™ tool due to potential technical implications, matched control groups based on 3D volumes of fetuses with normal CNS anatomy at similar gestational ages and similar maternal features were built as a reference. The analysis with paired t-test (Normality Test Shapiro–Wilk) and Wilcoxon Signed Rank Test showed a significantly higher need for manual plane adjustment in the abnormal collective (3.1 vs. 1.06 planes compared with the control group; *p* < 0.001). The same applied to the number of volumes required for a successful semiautomatic reconstruction using the 5D CNS+™ tool. We noticed a mean of 1.7 volumes in anomalous fetuses compared to 1.02 volumes (*p* < 0.001) in the matched control group. Based on our results, there was no significant difference (*p* = 0.677) found between the drop-out rates in both groups, with 0.1 in the abnormal group versus 0.03 in the control group.

## Discussion

In our study on 3D volume data sets of second- and third-trimester fetuses, we were able to demonstrate the potential of a standardized semiautomated approach of anatomic reconstruction of diagnostic CNS planes in the presence of congenital brain anomalies. Given the nonsatisfactory detection rates of CNS lesions in utero in an unselected population on the one hand and the diagnostic value of 3D ultrasound with simultaneous analysis in three orthogonal planes for advanced assessments of the fetal CNS (as presented herein) on the other hand, the question arises why most of the available data still rely on conventional two-dimensional ultrasound (2DUS). Following the recently revised ISUOG practice guidelines for sonographic examination of the fetal central nervous system, further assessment of additional diagnostic planes is mandatory in cases of a positive family history, congenital heart disease, suspected intrauterine infection, monochorionic twin gestation, abnormal genetic testing, or when an anatomic abnormality is suspected during routine or nuchal translucency scans [15]. As exemplified by an apparent enlargement of the ventricular system, a careful assessment of the entire intracranial anatomy is arbitrative, including the lateral, third, and fourth ventricles; (peri-)callosal region; thalami; germinal matrix region; and cerebellum, to rule out additional CNS anomalies (and extracranial defects). However, this, in turn, implicates attainment of numerous additional planes and views that primarily constitute a complete neurosonogram. Developing and maintaining the skills to perform a targeted anatomic survey in general, but fetal neurosonography in particular, requires an investment and an organized approach [16, 17].

A collaborative study analyzing the results from 11 international prenatal centers emphasized that 3D US of the fetal CNS constitutes an accurate and reliable method for the diagnosis of fetal brain malformations [8]. Assessment of prospective data from the Neurosofe-3D study demonstrated that acquisition of brain volumes following evidence-based guidelines is able to improve the quality of the volumes and visualization rates of the intracranial structures (satisfying evaluation of 91.5% structures in the axial plane, 81.8% in the coronal plane, and 89.9% in the sagittal plane) [18]. In light of very recent data from a complementary analysis of the INTERGROWTH-21 project cohort that investigated and measured fetal brain structures on ultrasound images extracted from 3D volumes of the fetal head, the importance of obtaining standardized planes using a volumetric approach has been underscored [19].

### Current research on volumetric assessment of CNS anatomy

In the last fifteen years, substantial efforts have been made toward a thorough volumetric assessment of fetal midbrain and hindbrain structures and their defects in utero (e.g., corpus callosum including pericallosal blood supply, 3rd ventricle and adjacent structures, posterior fossa and cerebellum, Sylvian fissure) [9, 20–26]. Spinelli et al. provided data on how the exact biometry of posterior fossa structures (vermian crest angle) can be obtained in a feasible and reproducible manner using 3D US [27]. Rodriguez-Sibaja and colleagues published international standards for fetal cerebellar growth and Sylvian fissure maturation using 3D ultrasound volumes from the same population-based project [28]. Qualitative and quantitative studies of cortical development evaluation using volume data sets clearly showed the potential of multiplanar assessment and confirmed 3D US as a robust diagnostic method [14, 29, 30]. Detailed prenatal judgment of even subtle changes in terms of cortical grading and sulci shape and depth is feasible [31–34], but it necessitates exact image alignment and, more strikingly, an awareness of the adequacy of planar image adjustment based on anatomical landmarks, as described in the current literature [29, 35].

In fact, quite a number of the studies analyzed volume information derived from transvaginally acquired 3D datasets, as these showed a higher image resolution (through US propagation via the sagittal suture and subsequent volume postprocessing) [36–38]. Three-dimensional TVS has been reported to have higher success rates than the 2D approach and is capable of multiplanar volume manipulation along the x-, y-, and z-axes to achieve high-quality images without requiring acquisition in the exact mid-sagittal plane [39, 40]. However, due to technical obstacles, such as the inability to manipulate the fetal head to an optimal scanning position or due to physical constraints that may limit the number of degrees of freedom of the US probe, transvaginal access to capture fetal CNS anatomy might be challenging or even impossible. Taking this into account, it becomes obvious that despite the somewhat lower resolution, an abdominal (rather than a transvaginal) approach using an axial acquisition plane is much more appropriate in a screening setting. Nevertheless, manual navigation through brain volume datasets necessitates a comprehensive understanding of fetal CNS architecture and a spatial sense of anatomic relations and hence is highly operator dependent, especially when CNS abnormalities are suspected. It has recently been shown that automated volumetric approaches efficiently enable rapid and standardized evaluation of the fetal brain in terms of basic examination [41–43] or reconstruction of an entire neurosonogram [12–14]. However, it remains widely unclear how these automatic techniques are of clinical use in delineating different CNS lesions by means of proper plane reconstruction and visualization of structural defects in detail.

### Clinical implications of the study findings

In all cases of our cohort affected by anomalies designated to the posterior fossa, including cerebellar lesions (Fig. 4), the diagnostic planes depicting the particular lesion were correctly reconstructed, which is in line with previous findings using the 5D CNS+™ algorithm [13]. The added value of orthogonally oriented sections displayed in a single template is exemplary and was demonstrated in both fetuses with rhombencephalosynapsis (RES) and cerebellar hypoplasia (Fig. 3). The common features of abnormal rhombencephalic differentiation as described previously can be tracked in a step-by-step manner [44–47]. While summarizing the abnormal anatomic hints of the fetus in Fig. 3, a Goméz-López-Hernandéz syndrome might be likewise suggested. Subtle anatomical measures that might point to concomitant mid- and hindbrain lesions such as tectum length/thickness and tegmental thickness or the absence of the sonolucent appearance of the aqueduct (best visualized in longitudinal and transcerebellar cutting sections) can also be quantified in detail as very recently reported [48]. Accordingly, occlusive lesions caused by obstruction of cerebrospinal fluid (CSF) pathways either to aqueductal stenosis or secondary to Chiari-II malformation were present in 7 cases and 21 cases, respectively, and were effectively displayed using 5D CNS+™ (Fig. 2). Depicting axial, coronal, and longitudinal simultaneously in a single template allows for a more comprehensive anatomic evaluation of the most likely underlying cause of the intracranial pathology. In Fig. 3, indirect signs of internal obstruction that were highly suggestive of AS and commonly seen on comparative MR imaging studies could be readily observed [49, 50]. Notably, in > 90% of cases with open spina bifida, there were no sonographic hints of discontinuation of CSF circulation downstream of the third ventricle. Birnbaum et al. introduced objective measures of the 3rd ventricle and its surrounding landmarks that have proven to be useful in diagnosing primary obstruction with high accuracy [20]. Prerequisites for assessment of the interthalamic adhesion diameter as the strongest proxy are precise midsagittal views through the mid- and hindbrain.

A detailed description of intracranial signs of neural tube defects (NTDs) from the first trimester onwards other than a small posterior fossa and the infratentorial descent of the brainstem and cerebellar tonsils (Chiari II malformation) has been proposed by several groups [51–54]. Very recently, the interpeduncular angle (IPA) was reported to be reduced in MR tomography in second- and third-trimester fetuses with dorsal dysraphism corresponding to a complete collapse of the interpeduncular cistern following severe hypotension in hindbrain herniation [55]. In our study cohort, a wide range of anatomical severities of intracranial pathology were encountered, encompassing different degrees of ventriculomegaly and hydrocephaly secondary to the observed changes within the Chiari II malformation spectrum.

As the correct plane for the calculation of the IPA is rather confined to an oblique cutting section traversing the eyes toward the occiput above the dislocated cerebellum, this measure could not be obtained accordingly.

In spite of the higher need for manual adjustment and numbers of volumes for proper semiautomatic reconstruction, 5D CNS+™ is an efficient and valuable method, but it must, however, be considered that similar to conventional 2D US, volume US has several technical limitations, particularly in advanced gestation (e.g., hardly defined edges, multiplicative speckle noise, and the only partially solved issue of estimating missing information in occluded areas caused by acoustic shadows, as seen in the analysis of the proximal parasagittal plane). Moreover, extraction of appropriate cutting sections from the fetal brain volumes depends on acoustic beam penetration and tissue impedance and is further influenced by the fact that most of the brain tissue has similar acoustic properties and impedance values [56]. A further limitation of our study is that the initial volume acquisition was made by operators with expertise in fetal US, which may introduce a certain bias on the success rates of 5D CNS+™ volume reconstruction. Another (methodologically) relevant fact has been stressed by Quarello et al., who stated that the choice of CSP instead of fornices as the anterior most structure (or pivotal point for automatic plane reconstruction) for cortical maturation assessment may lead to misinterpretation of the modification of the SF shape and is prone to a certain variation in cutting sections [35]. Although we generally agree, we believe that adherence to workflow-based volumetric approaches might considerably limit the extent of variation, imprecise diagnostic planes, and the need for manual plane adjustments.

The advent of newer imaging technologies, as discussed and illustrated herein, might further nourish the controversial debate regarding the role of fetal MRI in the diagnostic work-up of fetal brain anomalies (Fig. 5). Recent data from the European Neurosonography (ENSO) Working Group indicate that the incidence of an associated fetal anomaly in fetuses with a sonographic diagnosis of isolated mild or moderate ventriculomegaly (VM) or corpus callosum agenesis that was missed on ultrasound and detected only on fetal magnetic resonance imaging (MRI) is lower than that previously reported. These anomalies merely involve migration disorders and hemorrhage [57, 58]. Notably, no statistically significant differences were noted between the diagnostic accuracy of fetal neurosonography and fetal MRI for CC and CSP anomalies, NTDs, PFA, and PVM [59–61]. Accordingly, van der Knoop and colleagues stated that fetal MRI did not demonstrate any anomalies that were not seen on multiplanar neurosonography [62]. The complementary information of MR imaging in the evaluation of CNS pathologies might be of value when clarification of a US-identified lesion may advise pre- and postnatal management (including parental decision to terminate the pregnancy) or when feto-maternal conditions hamper detailed US examination [63, 64].

## Conclusions

In conclusion, although the assessment of fetal CNS anomalies continues to improve, the diagnostic potential of 3D ultrasound as a valuable tool in detailed structural analysis of the fetal brain has not been fully utilized. Despite the clinical value and advantages of 3D US its diagnostic potential remains underestimated, as many centers have not embraced this modality. The main reason for this dilemma and the limited uptake of 3D US might be attributed to the lack of standardization in the acquisition and postprocessing of volume data sets, constituting a major limiting step for the effective performance of 3D US. A workflow-based three-dimensional ultrasound approach, as reviewed herein, has been shown to reproducibly improve the assessment of both the normal and abnormal fetal CNS architecture and can be considered as an easy-to-apply screening and diagnostic tool in a clinical setting, since it enables a more refined diagnosis of most congenital malformations of the brain. Moreover, storage of 3D volume data sets enables offline review of the data volumes and facilitates, if needed, remote second opinions of specialists in the field. However, inexperienced examiners need to become familiar with the anatomy, rendered views, and spatial relationships of the fetal CNS and implement volume acquisition of fetal targets, giving them the opportunity to improve their daily routine in a convenient and time-saving manner.

In contrast to this rapid semiautomatic US technique, MRI consumes vast amounts of time (during which the main questions asked by parents could be addressed in detail by neuropediatricians) and resources and may overlook subtle anatomic anomalies such as a faulty cortical migration or intracranial hemorrhage (Fig. 5).

## Supplementary Information

**Additional file 1: Video file 1.** Resulting multiplanar orthogonal display following volume acquisition of the fetal head in a transthalamic position. After horizontal alignment of the falx (A and B plane) and subsequent application of the 5DCNS+ tool a nine-image template was automatically reconstructed showing a normal arrangement of the CNS anatomy at 21 gestational weeks.

**Additional file 2: Video file 2.** Abnormal CNS anatomy at 20 gestational weeks (massive ventricular enlargement, absent cavum septi pellucidi, agenesis of corpus callosum and rhombencephalosynapsis) simultaneously displayed in nine different views. The software allows manual adjustment of each particular plane for exact visualization of the altered anatomy.

## Data Availability

The datasets used and/or analyzed during the current study are available from the corresponding author on reasonable request.

## References

[1] Garne E, Loane M, Addor MC, Boyd PA, Barisic I, Dolk H (2010). Congenital hydrocephalus–prevalence, prenatal diagnosis and outcome of pregnancy in four European regions. Eur J Paediatr Neurol.

[2] Kinsner-Ovaskainen A, Morris JK, Garne E, Loane M, Lanzoni M (2020). European monitoring of congenital anomalies: JRC-EUROCAT report on statistical monitoring of congenital anomalies (2008–2017). In Luxembourg.

[3] EUROCAT registry. Prevalence charts and tables. European Commission. European Platform on Rare Disease Registration Web site. https://eu-rd-platform.jrc.ec.europa.eu/eurocat/eurocat-data/prevalence_en. Updated 28.05.2021. Accessed 30.06.2021, 2021.

[4] Morris JK, Wellesley DG, Barisic I (2019). Epidemiology of congenital cerebral anomalies in Europe: a multicentre, population-based EUROCAT study. Arch Dis Child.

[5] Van den Veyver IB (2019). Prenatally diagnosed developmental abnormalities of the central nervous system and genetic syndromes: a practical review. Prenat Diagn.

[6] Snoek R, Albers M, Mulder EJH (2018). Accuracy of diagnosis and counseling of fetal brain anomalies prior to 24 weeks of gestational age. J Matern Fetal Neonatal Med.

[7] Cardenas AM, Whitehead MT, Bulas DI (2020). Fetal Neuroimaging Update. Semin Pediatr Neurol.

[8] Rizzo G, Abuhamad AZ, Benacerraf BR (2011). Collaborative study on 3-dimensional sonography for the prenatal diagnosis of central nervous system defects. J Ultrasound Med.

[9] Bornstein E, Monteagudo A, Santos R (2010). Basic as well as detailed neurosonograms can be performed by offline analysis of three-dimensional fetal brain volumes. Ultrasound Obstet Gynecol.

[10] Salman MM, Twining P, Mousa H (2011). Evaluation of offline analysis of archived three-dimensional volume datasets in the diagnosis of fetal brain abnormalities. Ultrasound Obstet Gynecol.

[11] Maiz N, Alonso I, Belar M (2016). Three dimensional ultrasonography for advanced neurosonography (Neurosofe-3d): analysis of acquisition-related factors influencing the quality of the brain volumes. Prenat Diagn.

[12] Rizzo G, Aiello E, Pietrolucci ME, Arduini D (2016). The feasibility of using 5D CNS software in obtaining standard fetal head measurements from volumes acquired by three-dimensional ultrasonography: comparison with two-dimensional ultrasound. J Matern Fetal Neonatal Med.

[13] Rizzo G, Capponi A, Persico N (2016). 5D CNS+ software for automatically imaging axial, sagittal, and coronal planes of normal and abnormal second-trimester fetal brains. J Ultrasound Med.

[14] Welp A, Gembicki M, Rody A, Weichert J (2020). Validation of a semiautomated volumetric approach for fetal neurosonography using 5D CNS+ in clinical data from > 1100 consecutive pregnancies. Childs Nerv Syst.

[15] Malinger G, Paladini D, Haratz KK, Monteagudo A, Pilu GL, Timor-Tritsch IE (2020). ISUOG Practice Guidelines (updated): sonographic examination of the fetal central nervous system: Part 1: performance of screening examination and indications for targeted neurosonography. Ultrasound Obstet Gynecol.

[16] Malinger G, Lerman-Sagie T, Viñals F (2006). Three-dimensional sagittal reconstruction of the corpus callosum: fact or artifact?. Ultrasound Obstet Gynecol.

[17] Taipale P, Ammälä M, Salonen R, Hiilesmaa V (2003). Learning curve in ultrasonographic screening for selected fetal structural anomalies in early pregnancy. Obstet Gynecol.

[18] Maiz N, Tajada M, Rodríguez M (2021). Three-dimensional ultrasonography for advanced neurosonography (neurosofe-3D): Validation of a brain volume acquisition guideline. Acta Obstet Gynecol Scand.

[19] Napolitano R, Molloholli M, Donadono V (2020). International standards for fetal brain structures based on serial ultrasound measurements from Fetal Growth Longitudinal Study of INTERGROWTH-21(st) Project. Ultrasound Obstet Gynecol.

[20] Birnbaum R, Parodi S, Donarini G, Meccariello G, Fulcheri E, Paladini D (2018). The third ventricle of the human fetal brain: Normative data and pathologic correlation: a 3D transvaginal neurosonography study. Prenat Diagn.

[21] Leibovitz Z, Shkolnik C, Haratz KK, Malinger G, Shapiro I, Lerman-Sagie T (2014). Assessment of fetal midbrain and hindbrain in mid-sagittal cranial plane by three-dimensional multiplanar sonography: Part 1: comparison of new and established nomograms. Ultrasound Obstet Gynecol.

[22] Leibovitz Z, Shkolnik C, Haratz KK, Malinger G, Shapiro I, Lerman-Sagie T (2014). Assessment of fetal midbrain and hindbrain in mid-sagittal cranial plane by three-dimensional multiplanar sonography: Part 2: application of nomograms to fetuses with posterior fossa malformations. Ultrasound Obstet Gynecol.

[23] Miguelote RF, Vides B, Santos RF, Palha JA, Matias A, Sousa N (2011). The role of three-dimensional imaging reconstruction to measure the corpus callosum: comparison with direct mid-sagittal views. Prenat Diagn.

[24] Mittal P, Gonçalves LF, Kusanovic JP (2007). Objective evaluation of sylvian fissure development by multiplanar 3-dimensional ultrasonography. J Ultrasound Med.

[25] Pilu G, Segata M, Ghi T (2006). Diagnosis of midline anomalies of the fetal brain with the three-dimensional median view. Ultrasound Obstet Gynecol.

[26] Tonni G, Grisolia G, Sepulveda W (2014). Second trimester fetal neurosonography: reconstructing cerebral midline anatomy and anomalies using a novel three-dimensional ultrasound technique. Prenat Diagn.

[27] Spinelli M, Di Meglio L, Mosimann B, Di Naro E, Surbek D, Raio L (2019). The vermian-crest angle: a new method to assess fetal vermis position within the posterior fossa using 3-dimensional multiplanar sonography. Fetal Diagn Ther.

[28] Rodriguez-Sibaja MJ, Villar J, Ohuma EO (2021). Fetal cerebellar growth and Sylvian fissure maturation: international standards from Fetal Growth Longitudinal Study of INTERGROWTH-21(st) Project. Ultrasound Obstet Gynecol.

[29] Pooh RK, Machida M, Nakamura T (2019). Increased Sylvian fissure angle as early sonographic sign of malformation of cortical development. Ultrasound Obstet Gynecol.

[30] Rolo LC, Araujo Júnior E, Nardozza LM, de Oliveira PS, Ajzen SA, Moron AF (2011). Development of fetal brain sulci and gyri: assessment through two and three-dimensional ultrasound and magnetic resonance imaging. Arch Gynecol Obstet.

[31] Hahner N, Puerto B, Perez-Cruz M (2018). Altered cortical development in fetuses with isolated nonsevere ventriculomegaly assessed by neurosonography. Prenat Diagn.

[32] Paules C, Miranda J, Policiano C (2021). Fetal neurosonography detects differences in cortical development and corpus callosum in late-onset small fetuses. Ultrasound Obstet Gynecol.

[33] Pistorius LR, Stoutenbeek P, Groenendaal F (2010). Grade and symmetry of normal fetal cortical development: a longitudinal two- and three-dimensional ultrasound study. Ultrasound Obstet Gynecol.

[34] Quarello E, Stirnemann J, Ville Y, Guibaud L (2008). Assessment of fetal Sylvian fissure operculization between 22 and 32 weeks: a subjective approach. Ultrasound Obstet Gynecol.

[35] Quarello E, Guibaud L (2020). Prenatal sonographic assessment of Sylvian fissure operculization (SFO): importance of distinguishing between screening and diagnostic tools and selecting precise anatomical landmarks. Ultrasound Obstet Gynecol.

[36] Frisova V, Srutova M, Hyett J (2018). 3-D volume assessment of the corpus callosum and cerebellar vermis using various volume acquisition and post-processing protocols. Fetal Diagn Ther.

[37] Monteagudo A, Timor-Tritsch IE, Mayberry P (2000). Three-dimensional transvaginal neurosonography of the fetal brain: 'navigating' in the volume scan. Ultrasound Obstet Gynecol.

[38] Timor-Tritsch IE, Monteagudo A, Mayberry P (2000). Three-dimensional ultrasound evaluation of the fetal brain: the three horn view. Ultrasound Obstet Gynecol.

[39] Miguelote RF, Vides B, Santos RF, Matias A, Sousa N (2012). Feasibility and reproducibility of transvaginal, transabdominal, and 3D volume reconstruction sonography for measurement of the corpus callosum at different gestational ages. Fetal Diagn Ther.

[40] Wang PH, Ying TH, Wang PC, Shih IC, Lin LY, Chen GD (2000). Obstetrical three-dimensional ultrasound in the visualization of the intracranial midline and corpus callosum of fetuses with cephalic position. Prenat Diagn.

[41] Ambroise Grandjean G, Hossu G, Bertholdt C, Noble P, Morel O, Grangé G (2018). Artificial intelligence assistance for fetal head biometry: assessment of automated measurement software. Diagn Interv Imaging.

[42] Meng L, Zhao D, Yang Z, Wang B (2020). Automatic display of fetal brain planes and automatic measurements of fetal brain parameters by transabdominal three-dimensional ultrasound. J Clin Ultrasound.

[43] Pluym ID, Afshar Y, Holliman K (2021). Accuracy of automated three-dimensional ultrasound imaging technique for fetal head biometry. Ultrasound Obstet Gynecol.

[44] Cagneaux M, Vasiljevic A, Massoud M (2013). Severe second-trimester obstructive ventriculomegaly related to disorders of diencephalic, mesencephalic and rhombencephalic differentiation. Ultrasound Obstet Gynecol.

[45] Haratz KK, Oliveira Szejnfeld P, Govindaswamy M, Leibovitz Z, Gindes L, Severino M, Rossi A, Paladini D, Garcia Rodriguez R, Ben-Sira L, Borkowski Tillman T, Gupta R, Lotem G, Raz N, Hamamoto TENK, Kidron D, Arad A, Birnbaum R, Brussilov M, Pomar L, Vial Y, Leventer RJ, McGillivray G, Fink M, Krzeszowski W, Fernandes Moron A, Lev D, Tamarkin M, Shalev J, Har Toov J, Lerman-Sagie T, Malinger G (2021). Prenatal diagnosis of rhombencephalosynapsis: neuroimaging features and severity of vermian anomaly. Ultrasound Obstet Gynecol.

[46] Ishak GE, Dempsey JC, Shaw DW (2012). Rhombencephalosynapsis: a hindbrain malformation associated with incomplete separation of midbrain and forebrain, hydrocephalus and a broad spectrum of severity. Brain.

[47] Macé P, Ville Y, Bessière B, Quarello E (2021). Early diagnosis of rhombencephalosynapsis: the limits of intracranial translucency at first-trimester screening and a plea for assessment of aqueduct of Sylvius. Ultrasound Obstet Gynecol.

[48] Birnbaum R, Barzilay R, Brusilov M, Acharya P, Malinger G, Krajden HK (2022). Early second-trimester three-dimensional transvaginal neurosonography of fetal midbrain and hindbrain: normative data and technical aspects. Ultrasound Obstet Gynecol.

[49] Emery SP, Hogge WA, Hill LM (2015). Accuracy of prenatal diagnosis of isolated aqueductal stenosis. Prenat Diagn.

[50] Heaphy-Henault KJ, Guimaraes CV, Mehollin-Ray AR (2018). Congenital aqueductal stenosis: findings at fetal MRI that accurately predict a postnatal diagnosis. AJNR Am J Neuroradiol.

[51] Buisson O, De Keersmaecker B, Senat MV, Bernard JP, Moscoso G, Ville Y (2002). Sonographic diagnosis of spina bifida at 12 weeks: heading towards indirect signs. Ultrasound Obstet Gynecol.

[52] Liao Y, Wen H, Luo G (2021). Fetal open and closed spina bifida on a routine scan at 11 weeks to 13 weeks 6 days. J Ultrasound Med.

[53] Ushakov F, Sacco A, Andreeva E (2019). Crash sign: new first-trimester sonographic marker of spina bifida. Ultrasound Obstet Gynecol.

[54] Wertaschnigg D, Ramkrishna J, Ganesan S (2020). Cranial sonographic markers of fetal open spina bifida at 11 to 13 weeks of gestation. Prenat Diagn.

[55] Sepulveda F, Quezada F, Montoya F, Sepulveda W (2021). Interpeduncular angle: a new parameter for assessing intracranial hypotension in fetuses with spinal dysraphism. Prenat Diagn.

[56] Perez-Gonzalez J, Arámbula-Cosío F, Guzmán M (2018). Spatial compounding of 3-D fetal brain ultrasound using probabilistic maps. Ultrasound Med Biol.

[57] Di Mascio D, Sileo FG, Khalil A (2019). Role of magnetic resonance imaging in fetuses with mild or moderate ventriculomegaly in the era of fetal neurosonography: systematic review and meta-analysis. Ultrasound Obstet Gynecol.

[58] Sileo FG, Di Mascio D, Rizzo G (2021). Role of prenatal magnetic resonance imaging in fetuses with isolated agenesis of corpus callosum in the era of fetal neurosonography: a systematic review and meta-analysis. Acta Obstet Gynecol Scand.

[59] Paladini D, Quarantelli M, Sglavo G (2014). Accuracy of neurosonography and MRI in clinical management of fetuses referred with central nervous system abnormalities. Ultrasound Obstet Gynecol.

[60] Tanacan A, Ozgen B, Fadiloglu E, Unal C, Oguz KK, Beksac MS (2020). Prenatal diagnosis of central nervous system abnormalities: Neurosonography versus fetal magnetic resonance imaging. Eur J Obstet Gynecol Reprod Biol.

[61] Tercanli S, Prüfer F (2016). Fetal neurosonogaphy: ultrasound and magnetic resonance imaging in competition. Ultraschall Med.

[62] van der Knoop BJ, Zonnenberg IA, Verbeke J (2020). Additional value of advanced neurosonography and magnetic resonance imaging in fetuses at risk for brain damage. Ultrasound Obstet Gynecol.

[63] Masselli G, Vaccaro Notte MR, Zacharzewska-Gondek A, Laghi F, Manganaro L, Brunelli R (2020). Fetal MRI of CNS abnormalities. Clin Radiol.

[64] Paladini D, Malinger G, Birnbaum R (2021). ISUOG Practice Guidelines (updated): sonographic examination of the fetal central nervous system: Part 2: performance of targeted neurosonography. Ultrasound Obstet Gynecol.

[65] Abuhamad AZ (2005). Standardization of 3-dimensional volumes in obstetric sonography: a required step for training and automation. J Ultrasound Med.

[66] Dall'Asta A, Paramasivam G, Basheer SN, Whitby E, Tahir Z, Lees C (2019). How to obtain diagnostic planes of the fetal central nervous system using three-dimensional ultrasound and a context-preserving rendering technology. Am J Obstet Gynecol.

